# Heterogeneity in *ess* transcriptional organization and variable contribution of the Ess/Type VII protein secretion system to virulence across closely related *Staphylocccus aureus* strains

**DOI:** 10.1111/mmi.12707

**Published:** 2014-07-30

**Authors:** Holger Kneuper, Zhen Ping Cao, Kate B Twomey, Martin Zoltner, Franziska Jäger, James S Cargill, James Chalmers, Magdalena M van der Kooi-Pol, Jan Maarten van Dijl, Robert P Ryan, William N Hunter, Tracy Palmer

**Affiliations:** 1 Division of Molecular Microbiology, College of Life Sciences, University of DundeeDundee, UK; 2 Division of Biological Chemistry and Drug Discovery, College of Life Sciences, University of DundeeDundee, UK; 3 School of Microbiology, Biosciences Institute, University College CorkCork, Ireland; 4 Department of Medical Microbiology, University of Groningen, University Medical Center GroningenHanzeplein 1, P.O. Box 30001, 9700 RB, Groningen, The Netherlands

## Abstract

The Type VII protein secretion system, found in Gram-positive bacteria, secretes small proteins, containing a conserved W-x-G amino acid sequence motif, to the growth medium. *Staphylococcus aureus* has a conserved Type VII secretion system, termed Ess, which is dispensable for laboratory growth but required for virulence. In this study we show that there are unexpected differences in the organization of the *ess* gene cluster between closely related strains of *S*. *aureus*. We further show that in laboratory growth medium different strains of *S*. *aureus* secrete the EsxA and EsxC substrate proteins at different growth points, and that the Ess system in strain Newman is inactive under these conditions. Systematic deletion analysis in *S*. *aureus* RN6390 is consistent with the EsaA, EsaB, EssA, EssB, EssC and EsxA proteins comprising core components of the secretion machinery in this strain. Finally we demonstrate that the Ess secretion machinery of two *S*. *aureus* strains, RN6390 and COL, is important for nasal colonization and virulence in the murine lung pneumonia model. Surprisingly, however, the secretion system plays no role in the virulence of strain SA113 under the same conditions.

## Introduction

Most bacteria secrete proteins into their external environments, where they play essential roles in nutrient acquisition, colonization and host interactions. Gram-negative bacteria may elaborate any of six different protein secretion systems (named Type I through Type VI) to move proteins across their double-membrane cell envelopes, in either a single step, or by a two-step mechanism (Desvaux *et al.*, 2009). Protein secretion systems that operate by a two-step mechanism first rely on the translocation of proteins across the inner membrane by either the general secretory (Sec) or twin arginine translocase (Tat) machineries, before they mediate the secretion of substrates across the outer membrane (e.g. Pugsley, 1993; Voulhoux *et al.*, 2001).

Protein secretion in Gram-positive bacteria is, in most cases, a simpler process because these organisms (with the exception of the didermic Mycobacterial and Corynebacterial species) are bounded by a single membrane. The Sec and Tat pathways, to which proteins are targeted by the presence of cleavable signal peptides at their N-termini, directly secrete extracellular proteins in these bacteria (e.g. Sibbald *et al.*, 2006; Widdick *et al.*, 2006; Goosens *et al.*, 2014). In addition, a secretion system that has, to date, only been experimentally described in Gram-positive bacteria, variously termed the ESX, Ess or Type VII protein secretion system, also serves to translocate proteins to the extracellular environment. This secretion system was first described in pathogenic mycobacteria where it secretes two T cell antigens, ESAT-6 (early secreted antigenic target, 6 kDa) and CFP-10 (culture filtrate protein 10 kDa), now renamed EsxA and EsxB, respectively, and was shown to be essential for virulence (Hsu *et al.*, 2003; Pym *et al.*, 2003; Stanley *et al.*, 2003). This secretion system has variously been shown to function in other actinobacterial species, including non-pathogenic members (Abdallah *et al.*, 2006; Akpe San Roman *et al.*, 2010; Fyans *et al.*, 2013).

A related secretion system is found in bacteria of the low-GC *Firmicutes* phylum. It has been best characterized in the opportunistic human and animal pathogen *Staphylococcus aureus*. The relationship between the *S. aureus* secretion system and the Mycobacterial ESX machineries is limited, with only two types of conserved components. The first is an ATPase of the FtsK/SpoIIIE protein family, while the second is the presence of one or more of the secreted EsxA/EsxB proteins (Pallen, 2002). EsxA and EsxB are small acidic proteins of the WXG100 superfamily that are structurally organized as a helical hairpin with a conserved Trp–Xaa–Gly (WXG) motif that localizes in a loop between the two α-helices (e.g. (Renshaw *et al.*, 2005; Sundaramoorthy *et al.*, 2008). In *Mycobacteria*, EsxA and EsxB form tight heterodimers that are co-dependent on each other for secretion by the Type VII system (Renshaw *et al.*, 2002; Stanley *et al.*, 2003; Champion *et al.*, 2006), whereas in *S. aureus* and related bacteria EsxA forms homodimers (Sundaramoorthy *et al.*, 2008; Shukla *et al.*, 2010; Anderson *et al.*, 2013). Other essential secretion components are non-conserved (at least at the amino acid sequence level) between actinobacterial and *Firmicutes* systems, leading to them being designated as ESX (actinobacteria) and Ess (*Firmicutes*) secretion systems respectively (Burts *et al.*, 2005; 2008,; Bitter *et al.*, 2009).

In *S. aureus* the Ess system has been shown to contribute to virulence in a mouse model of abscess formation. Mutations in the *S. aureus* Newman strain where any of *esxA*, *esxB* or *essC* (which encodes the FtsK/SpoIIIE family ATPase) were inactivated resulted in a significant reduction in cfu recovered from the livers and kidneys of mice that had been retro-orbitally injected with these strains (Burts *et al.*, 2005). It was later shown that EsxC (formerly EsaC), a conserved *S. aureus* protein, is a substrate of the Ess machinery that has a small role in abscess formation but a more significant role during long-term persistence of abscesses (Burts *et al.*, 2008). Recently EsxD was identified as a further secreted substrate (Anderson *et al.*, 2013). The precise function of any of the *S. aureus* Ess substrate proteins remains to be elucidated.

**Figure 1:**
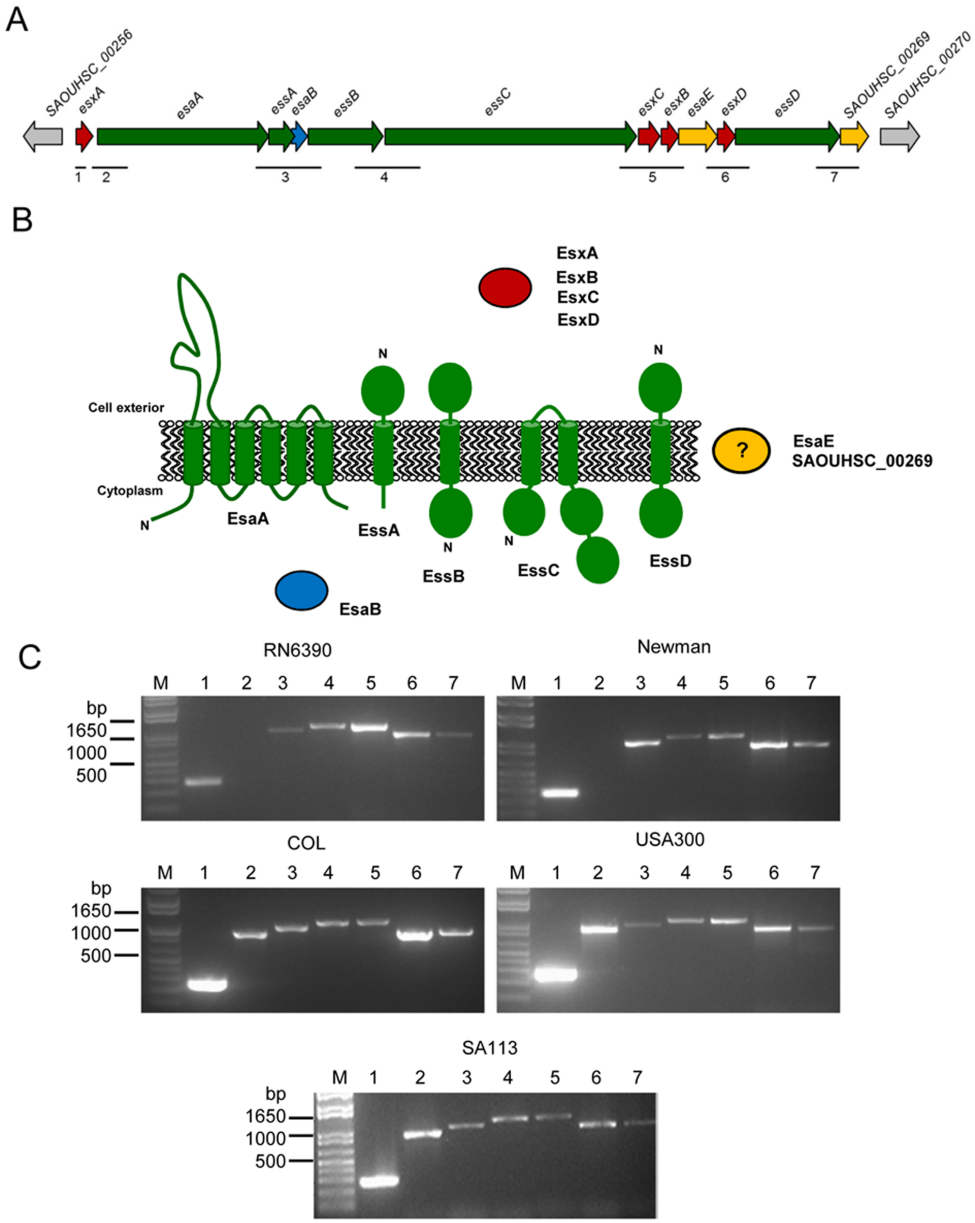
Organization of the *ess* locus in different strains of *S*. *aureus*. A. Schematic representation of the *S. aureus ess* locus derived from the NCTC8325 genome sequence. The region from the start of *esxA* to the end of *SAOUHSC_00269* covers approximately 14 kb and is almost 100% identical between the RN6390, Newman, COL and USA300 strains (only two nucleotide differences over this region). Genes encoding secreted substrates are coloured in red, membrane components in green, cytoplasmic components in blue and unknown components in orange. Putative unrelated genes are shaded in grey. Note that the sizes of the intergenic regions are as follows: *SAOUHSC_00256*-*esxA*, 247 bp; *esxA*-*esaA*, 83 bp; *esaA*-*essA*, −1 bp; *essA*-*esaB*, −68 bp; *esaB*-*essB*, 12 bp; *essB*-*essC*, 21 bp; *essC*-*esxC*, 29 bp; *esxC*-*esxB*, 15 bp; *esxB*-*esaE*, −1 bp; *esaE*-*esxD*, −1 bp; *esxD*-*essD*, 9 bp; *essD*-*SAOUHSC_00269*, 10 bp; *SAOUHSC_00269-SAOUHSC_00270*, 207 bp. B. Schematic representation of subcellular location and predicted topologies of the proteins encoded at the *ess* locus (not to scale). Shading as in part A. C. RT-PCR analysis of mRNA isolated from each of the five different strains, using primer pairs listed in Table S1. The expected sizes for PCR products 1–7 are 272, 953, 1023, 1153, 1168, 959 and 946 bp respectively.

The *S. aureus* Ess secretion system is encoded within the *ess* gene cluster and transcription of *esxA*, the first gene of the cluster, has been shown to be subject to complex regulation by the alternative sigma factor σ^B^ (Schulthess *et al.*, 2012). In the *S. aureus* strain Newman background, *esxA* appears to be monocistronic and is not co-transcribed with the downstream *ess* genes (Schulthess *et al.*, 2012). However, transposon mutagenesis has confirmed that each of EssA, EssB, EssC, which are encoded downstream of *esxA* in the *ess* cluster (Fig. 1A) are essential for the secretion of EsxA or EsxB (Burts *et al.*, 2005). The EssA, EssB and EssC proteins are membrane-bound (Burts *et al.*, 2005; Chen *et al.*, 2012; Zoltner *et al.*, 2013b) and probably form the membrane-embedded secretion apparatus. Two further membrane proteins are also encoded at the *ess* locus. Of these EsaA is reported to have no role in EsxA secretion (Burts *et al.*, 2005), while loss of EsaD (recently re-named EssD) reduces, but does not abolish, the export of EsxA (Anderson *et al.*, 2011).

In this study we have examined the organization of the *ess* gene cluster in a range of *S. aureus* strain backgrounds. Our results indicate that there are unexpected differences in the organization of the cluster, with the *esxA* gene being clearly co-transcribed with downstream *ess* genes in the COL, USA300 and SA113 strains, but transcribed as a monocistronic gene in the RN6390 and Newman strains. In the RN6390 and COL strains, EsxA and EsxC secretion could be detected throughout the growth phase, with substantial levels of extracellular protein accumulating from mid-logarithmic growth onwards. Systematic deletion analysis in the RN6390 strain background confirmed prior observations that *essA*, *essB* and *essC* were required for the secretion of EsxA, but surprisingly we also show that *esaA* and *esaB* are essential for secretion of EsxA and EsxC. Finally we show that the Ess secretion machinery of two *S. aureus* strains (RN6390 and COL) but not a third (SA113) is important for murine nasal colonization and virulence in the murine lung pneumonia model.

## Results

### The *esxA* gene is co-transcribed with downstream genes in *S*. *aureus* COL, USA300 and SA113 strains, but not in RN6390 or Newman

The *ess* gene cluster (Fig. A) has been reported to comprise at least 11 genes, several of which have essential or accessory roles in the secretion of Ess substrate proteins (Burts *et al.*, 2005; 2008; Anderson *et al.*, 2011). Previous work using the *S. aureus* Newman strain showed that *esxA* was a monocistronic gene, but the arrangement of the downstream genes was not examined (Schulthess *et al.*, 2012). We therefore decided to examine whether any of these genes were co-transcribed.

To this end, mRNA was isolated from each of five different *S. aureus* strains during exponential growth in TSB medium (OD_600_ of 2.0). The strains we selected, RN6390, Newman, USA300, SA113 and COL are all relatively closely related strains of *S. aureus* from clonal complex 8 (CC8). Strains RN6390, Newman, USA300 and SA113 are of the same sequence type, ST8, whereas COL is of sequence type 250 within CC8 (Herbert *et al.*, 2010). Oligonucleotide primer pairs were designed that either primed within the *esxA* gene (primer pair 1), that spanned the 83 bp intergenic region between *esxA* and *esaA*, or that spanned intergenic regions between the additional downstream genes (primer pairs 3 – 7; Fig. 1A, Table S1). For strains Newman and RN6390, primers amplifying within *esxA* gave a product of the expected size; however, no product was obtained using primers that spanned between *esxA* and *esaA* (Fig. 1C), confirming previous findings with Newman that *esxA* is a monocistronic gene (Schulthess *et al.*, 2012). Unexpectedly, however, the same primer pairs amplified a clear product of the expected size from the mRNA of strains COL, USA300 and SA113. Thus it can be concluded that the *esxA* and *esaA* genes are part of the same transcriptional unit in these three strains, and that there is heterogeneity in the transcriptional organization of the *ess* gene cluster between different strains of *S. aureus*.

Primer pairs 3–7 gave products of the expected sizes for mRNA isolated from all five *S. aureus* strains, strongly indicating the presence of a single transcript spanning from *esaA* to *SAOUHSC_00269*, for strains Newman and RN6390, and from *esxA* to *SAOUHSC_00269*, for strains COL, USA300 and SA113. These findings suggest that the uncharacterized gene *SAOUHSC_00269* is associated with Ess secretion.

### A promoter is present in the *esxA*-*esaA* intergenic region in strain RN6390

**Figure 2:**
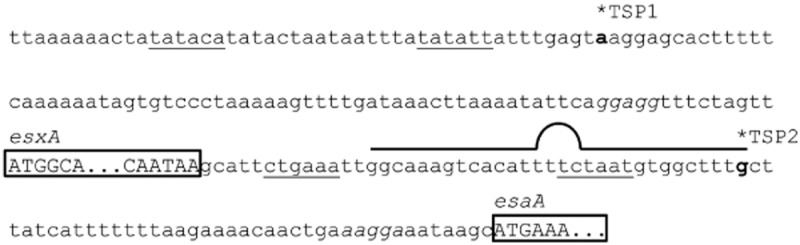
A transcription start site in the *esxA*-*esaA* intergenic region of strain RN6390. DNA sequence covering the promoter region and transcriptional start site of the *esxA* gene through to the start of *esaA*. Note that the sequence of this region is identical between all strains used in this study. Transcriptional start points (indicated TSP1 and TSP2), mapped by 5′-RACE, are indicated in bold. TSP1 was identified from strains RN6390 and USA300, whereas TSP2 was identified from RN6390 only. Putative −35/−10 regions are underlined, the coding regions for *esxA* and *esaA* are shown in upper case letters and boxed (*esxA* is truncated to save space), and putative Shine–Dalgarno sequences are given in italics. A predicted rho-independent terminator for the *esxA* gene is indicated above the sequence.

To confirm the findings from the RT PCR experiments, 5′-RACE was used to identify transcription start sites within the *esxA*-*esaA* intergenic region and upstream of *esxA*, using an oligonucleotide that primes within *esaA*, or within *esxA* respectively. We focused on strains RN6390 and USA300, representing the two different transcriptional organizations of the *ess* operon, and sampled at two time points during exponential growth in TSB medium (OD_600_ of 1.0 and 2.0 respectively). When mRNA from RN6390 was used as template, a transcriptional start site within the *esxA*-*esaA* intergenic region was identified (labelled TSP2 in Fig. 2). This start site is downstream of a likely promoter sequence showing a reasonable match to the −35 and −10 consensus sequences. This transcriptional start site was not identified when mRNA from USA300 was used, instead the sequence read continued into the upstream *esxA* gene. Using a primer that primes within *esxA*, the same transcriptional start site (TSP1 in Fig. 2) was identified from both of the mRNA samples. This is the same start site for *esxA* transcription identified by Schulthess *et al.* (2012).

It is interesting to note that there is a potential rho-independent terminator sequence in the *esxA-esaA* intergenic region, overlapping with the transcriptional start site and putative promoter for *esaA*. The same sequence is found in all five strains, and although it presumably acts as an effective terminator in strains Newman and RN6390, it must allow at least partial readthrough in the other strains examined here.

### The *esxA* gene is overexpressed compared to other genes in the *ess* cluster

We next examined whether the two different transcriptional organizations of the *ess* clusters affected the ratio between *esxA* transcript level and the levels of downstream transcripts. For these experiments, mRNA was isolated from strains RN6390, USA300 and COL when the culture optical density at 600 nm (OD_600_) reached 1.0 and 2.0 respectively. Reverse transcription of the mRNA followed by quantitative PCR, using the relative standard curve method, was undertaken to determine the relative levels of the *ess* genes *esxA*, *esaA*, *essC* and *esxB*, using the 16s rRNA gene as the endogenous control in these experiments. The relative expression levels were subsequently normalized, against *essC*, which was set to a relative expression level of 1 in each case.

**Figure 3:**
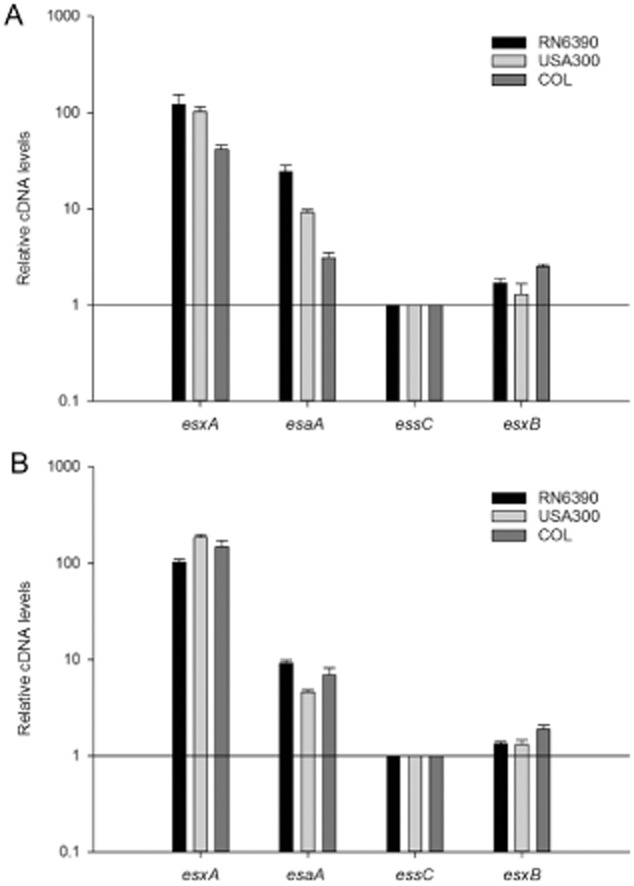
The *esxA* gene is overexpressed compared to *esaA*, *essC* and *esxB*. mRNA isolated from the indicated strains that had been grown aerobically in TSB medium to an OD_600_ of 1 (A) or 2 (B) was reverse transcribed into cDNA for use in quantitative RT-qPCR experiments. Relative cDNA of *esxA*, *esaA* and *esxB*, calibrated in relation to *essC* levels, were calculated by the relative standard curve method (Larionov *et al.*, 2005), using 16S cDNA as endogenous control.

As shown in Fig. 3, *esxA* is by far the most highly expressed of the *ess* genes tested, at an expression level approximately 100 times higher than that of *essC* for all three strains. The *esaA* gene is also more highly expressed than *essC*, being up to around 10 times more highly expressed, while *esxB* is expressed to similar levels as *essC* in all three strains. We conclude that despite the different mechanisms for transcriptional control of the *ess* genes in different *S. aureus* strains they ultimately give similar transcript level ratios among the encoded genes.

### The Ess secretion system is active in different *S*. *aureus* strains

The experiments described above show that mRNA encoding Ess secretion system components is expressed in *S. aureus* strains cultured in the laboratory growth medium Trypticase Soy Broth (TSB). To determine whether the secretion system is active under these growth conditions, each strain was cultured under similar growth conditions and samples were withdrawn when the culture OD_600_ reached 1.0, 2.0, 3.0 or 5.0. The withdrawn samples were centrifuged to separate the cells from the culture supernatant, and each fraction was analysed by Western blotting with antibodies to each of the two Ess-secreted proteins EsxA and EsxC, and the cytoplasmic marker protein TrxA.

**Figure 4:**
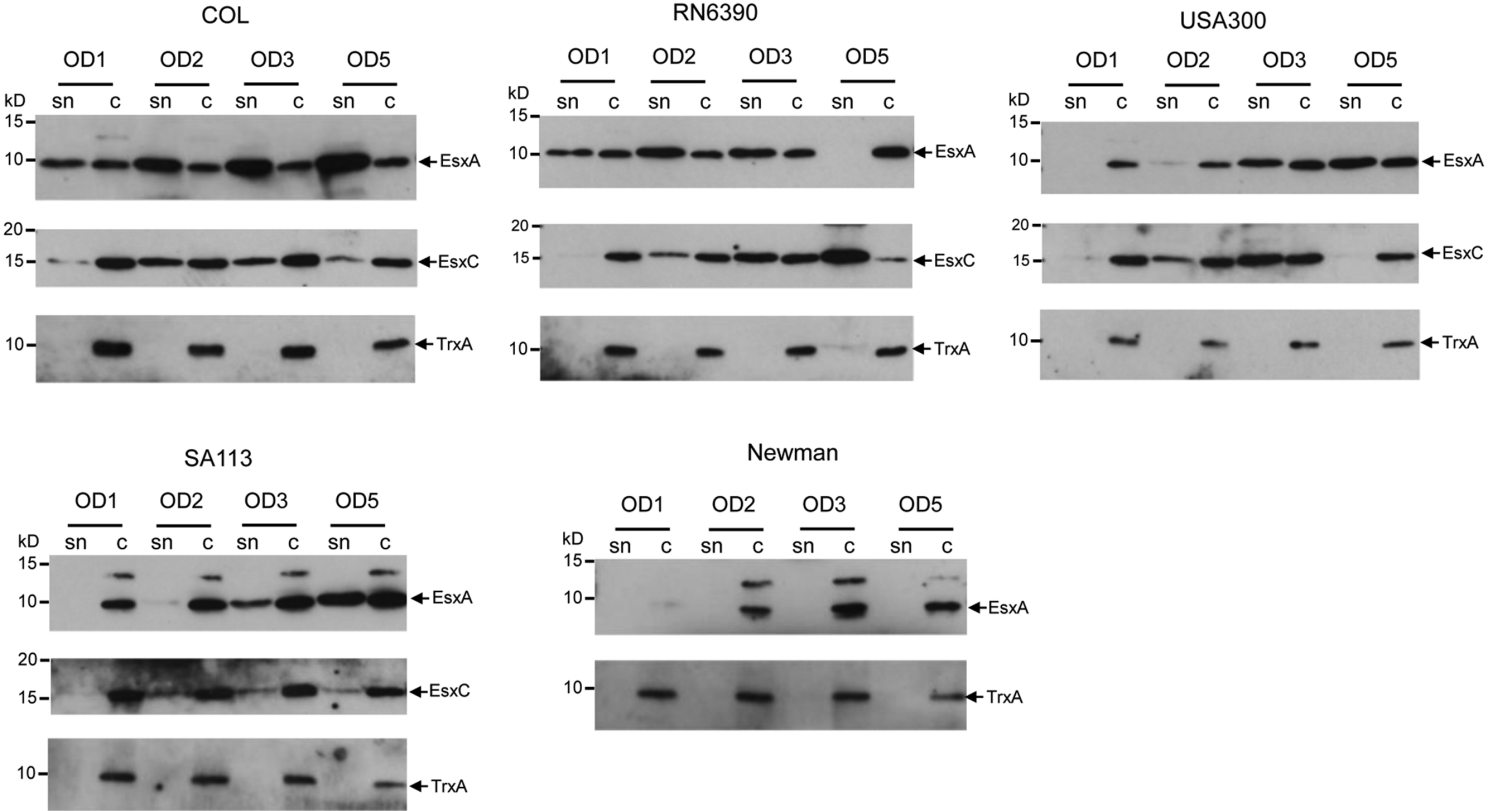
The Ess secretion system is active in different *S*. *aureus* strains. The indicated *S*. *aureus* strains were cultured in TSB medium, and once the OD_600_ of the culture reached each of 1, 2, 3 or 5 an aliquot was removed and centrifuged to give the culture supernatant (sn) and cellular (c) fractions. Samples were separated on 15% bis-Tris gels and immunoblotted using anti-EsxA, EsxC or TrxA antisera. For the EsxA blots, 7.5 μl of culture supernatant and 5 μl of cells adjusted to an OD_600_ of 0.25 were loaded (5 μl of OD_600_ 1 cells for Newman and SA113). For the TrxA blots, twice the amount of supernatant and cells samples as described for the EsxA blots were used. For the EsxC blots, TCA precipitated supernatant equivalent to 100 μl culture supernatant and 10 μl of cells adjusted to an OD_600_ of 1 were loaded.

As shown in Fig. 4, secretion of both of the Ess substrates tested could be detected for strains RN6390, USA300, COL and SA113. Ess secretion activity of USA300 grown in TSB medium has also been reported previously (Anderson *et al.*, 2013). For strains COL and RN6390 secretion of EsxA and EsxC could be detected at the earliest growth point tested, whereas secretion of these proteins was not detected for USA300 until the cells had reached an OD_600_ of 2, and for SA113 secretion was only clearly detectable when cells reached an OD_600_ of 3 or above. It should be noted that the presence of EsxA was detected on the surface of cells in shaving experiments of COL harvested in early exponential phase (Dreisbach *et al.*, 2010; Solis *et al.*, 2010). At the latest growth point tested, EsxA (but not EsxC) had disappeared from the culture supernatant of RN6390, although it was still present in the supernatants of the other three strains. It has been reported that RN6390 is deregulated for the production of extracellular protease which may account for the loss of EsxA from the supernatant at high cell density (Karlsson and Arvidson, 2002; Rigoulay *et al.*, 2005). Indeed, we did note the presence of an additional smaller form of EsxA in the supernatant fractions of RN6390 (but not of other strains) with some batches of our polyclonal EsxA antiserum (not shown).

By contrast with the other four strains, no secretion of EsxA was detectable for strain Newman at any of the growth points examined. Some EsxA antigen was detected at OD_600_ of 2 and above, but this was found exclusively in the cellular fraction. We were unable to detect any clear signal for EsxC in either the supernatant or cellular fractions (not shown) – this is consistent with a previous study which showed that EsxC was repressed when Newman was grown in broth (Burts *et al.*, 2008).

### Deletion analysis confirms that EssA, EssB and EssC are essential to support Ess-dependent secretion in strain RN6390

**Figure 5:**
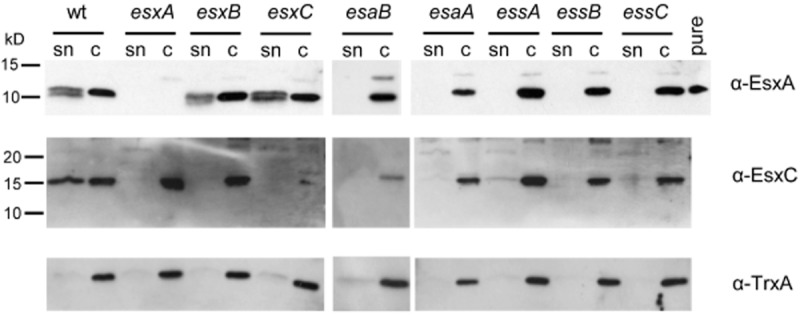
EsxA and EsxC are not detected in culture supernatants if *esaA*, *esaB*, *essA*, *essB*, or *essC* are deleted in the RN6390 strain background. The RN6390 wild-type (wt) or isogenic deletion strains, as indicated, were cultured in TSB medium until an OD_600_ of 2 was reached. Samples were withdrawn and separated into culture supernatant (sn) and cellular (c) fractions. An equivalent of 200 μl of culture supernatant and 10 μl of resuspended cell sample adjusted to a calculated cell density of OD 1 was loaded in each lane, samples were separated on 15% bis-Tris gels and immunoblotted with anti-EsxA, anti-EsxC or anti-TrxA antibodies. For the EsxA immunoblot, 1 ng of purified EsxA protein (pure) was loaded as control. Molecular weight markers are indicated to the left of the blot.

To identify genes encoded at the *ess* locus that are essential for Ess secretion system activity, we constructed in-frame deletions of each of *esaA*, *esaB*, *essA*, *essB*, *essC*, *esxA*, *esxB* and *esxC* in *S. aureus* strain RN6390. We first confirmed that each of the individual deletion strains did not have a polar effect on downstream genes by blotting for the presence of the secretion substrate EsxC (Fig. 5) and/or the membrane components EsaA, EssB and EssC (Fig. S2). We then tested the effect of each individual deletion on the presence of Ess substrates EsxA and EsxC in the culture supernatant.

EssA and EssB are monotopic membrane proteins (Fig. 1B) that are conserved components encoded at *ess* loci in Firmicutes (e.g. Burts *et al.*, 2005; Baptista *et al.*, 2013). Previous studies in the Newman strain background reported that transposon insertions in either *essA* or *essB* abolished secretion of EsxA (Burts *et al.*, 2005). In agreement with this, it can be seen (Fig. 5) that in-frame deletion of either *essA* or *essB* resulted in a complete absence of EsxA from the culture supernatant. However, in the Newman background insertional inactivation of either of these genes resulted in a destabilization of EsxA and EsxB (Burts *et al.*, 2005), whereas in RN6390 (Fig. 5), as in USA300 (Anderson *et al.*, 2013), EsxA protein is clearly detectable in the cellular fraction, even when the Ess system is inactivated. Analysis of EsxC localization showed a similar pattern, i.e. that deletion of *essB* resulted in no detectable secretion of EsxC, and that deletion of *essA* also resulted in a very severe EsxC secretion defect, although we routinely always detect a very low level of EsxC in the supernatant fraction of this strain. Again it should be noted that, similar to EsxA, cellular EsxC appeared stable even when the Ess system was inactivated.

EssC is a bitopic membrane protein that is conserved among the Ess and ESX secretion systems. Sequence and structural analysis indicates that the *Firmicutes* proteins have an N-terminal cytoplasmic region that adopts a tandem forkhead-associated domain fold and two iterations of the FtsK/SpoIIIE family domain in the C-terminal region that contain putative ATP binding P-loop motifs (Tanaka *et al.*, 2007). Wherever examined, the EssC-like proteins have been shown to be essential for Ess secretion (e.g. Burts *et al.*, 2005; Garufi *et al.*, 2008; Baptista *et al.*, 2013), and indeed in-frame deletion of *essC* in *S. aureus* RN6390 leads to total absence of EsxA and EsxC from the culture supernatant (Fig. 5).

To determine where the EsxA and EsxC proteins were localized when the secretion system was inactivated, we fractionated cells of the *essA* and *essC* mutant strains, along with the wild-type into the cell wall, membrane and cytoplasmic fractions. These samples were then probed with anti-EsxA and anti-EsxC antisera, along with antisera to the control proteins thioredoxin A (TrxA), protein A (Spa) or sortase A (SrtA). In the wild-type strain, EsxA and EsxC were detected largely in the cytoplasmic and supernatant samples, although some antigen was detected in the cell wall and traces of EsxC were also present in the membrane fraction. By contrast, in the *essC* mutant strain EsxA and EsxC were detected exclusively in the cytoplasmic fraction, confirming that EssC is essential for secretion of these proteins across the cytoplasmic membrane. Fractionation of the *essA* strain gave a slightly different result. Although EsxA was found exclusively in the cytoplasmic fraction in the absence of EssA, some EsxC protein could be detected in the cell wall and membrane fraction in the *essA* mutant. This is consistent with the results presented in Fig. 4, where low levels of EsxC were detected in the supernatant of the *essA* strain. We conclude that although clearly important, EssA is not absolutely essential for secretion of EsxC.

### Deletion analysis reveals an essential role for EsaA and EsaB in the export or extracellular stability of Ess substrates in strain RN6390

**Figure 6:**
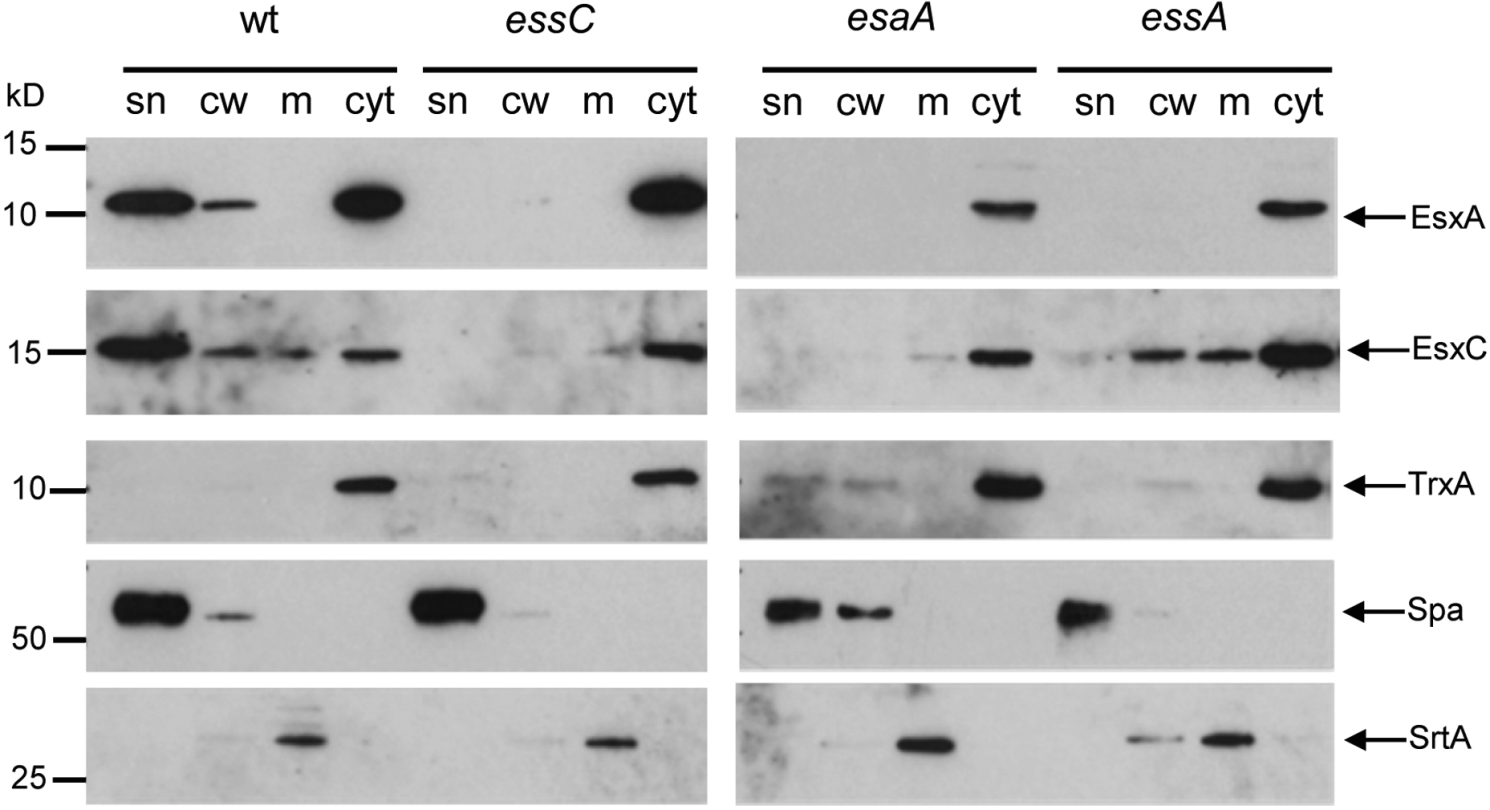
Subcellular location of EsxA and EsxC in RN6390 and isogenic *esaA*, *essA* and *essC* mutant strains. The RN6390 wild-type (wt) or isogenic deletion strains, as indicated, were cultured in TSB medium until an OD_600_ of 2 was reached. The cells were spun down and the supernatant (sn) was retained as the secreted protein fraction. The cell pellets were subsequently fractionated into cell wall (cw), membrane (m) and cytoplasmic (cyt) fractions as described in *Experimental procedures*. An equivalent of 100 μl of culture supernatant and cell wall, membrane and cytoplasmic samples corresponding to the respective fractions from 10 μl of cells adjusted to an OD_600_ of 1 were separated on bis-Tris gels and immunoblotted using the anti-EsxA or EsxC antisera, or control antisera raised to TrxA (cytoplasmic protein), protein A (Spa, cell wall) or sortase A (SrtA, membrane).

EsaA is a polytopic membrane protein with a long extracellular loop that has been shown through protease shaving experiments to extend to the surface of *S. aureus* (Dreisbach *et al.*, 2010). The homologous protein in *Bacillus subtilis*, YueB, is also surface-exposed and serves as a receptor for phage SPP1 infection (Sao-Jose *et al.*, 2004; 2006,). It has previously been reported in the Newman strain background that a transposon insertion in *esaA* had no effect on EsxA and EsxB secretion (Burts *et al.*, 2005). Interestingly, however, we noted that deletion of *esaA* completely abolished secretion of EsxA and EsxC in strain RN6390. It is unlikely that the secretion defect seen with the RN6390 *esaA^−^* strain arose due to polar downstream effects because (i) the EssB and EssC proteins that are encoded immediately downstream of *esaA* are clearly detectable in the *esaA* mutant strain (Fig. S2), and (ii) re-introduction of plasmid-encoded his-tagged EsaA into the *esaA* mutant background restored EsxA secretion (Fig. S3). We therefore conclude that in RN6390, EsaA is an essential component of the Ess secretion machinery. It should be noted that the *B. subtilis* EsaA homologue, YueB, which is encoded within the *B. subtilis ess* gene cluster, is similarly also essential for the secretion of *B. subtilis* EsxA (YukE) (Baptista *et al.*, 2013). Subcellular fractionation of the *esaA* strain showed that EsxA and EsxC were located almost exclusively in the cytoplasm (Fig. 6).

We also examined the roles of soluble Ess-encoded components on secretion of EsxA and EsxC. EsaB is a small (93 aa) protein that is structurally closely related to ubiquitin, although it lacks the conserved C-terminal double glycine motif (van den Ent and Lowe, 2005). It has been reported to act as a negative regulator for EsxC in *S. aureus* Newman and EsxC could only be detected when *esaB* was deleted. EsxC antigen could be detected in other strains of *S. aureus*, but again in the USA300 background deletion of *esaB* resulted in upregulation of EsxC levels. In each case loss of *esaB* did not affect secretion of EsxC to the growth medium (Burts *et al.*, 2008). Deletion of *esaB* in strain RN6390 resulted in an unexpected phenotype, because it abolished detectable secretion of EsxA and EsxC, indicating that it is essential for Ess secretion system activity in this strain background. It should be noted that a homologue of *S. aureus* EsaB (YukD) is also encoded within the *B. subtilis ess* gene cluster and is likewise essential for the secretion of EsxA (Baptista *et al.*, 2013). Thus, an essential role for EsaB in Ess secretion appears to be at least partially conserved.

Previous reports have indicated that in strain Newman the presence of EsxB is essential for the stability of EsxA (Burts *et al.*, 2005). However in RN6390, loss of *esxB* does not affect the stability or secretion of EsxA (Fig. 5). This is also in contrast to reports of the interdependence of EsxA and EsxB for secretion by the ESX machinery in *Mycobacteria* (Champion *et al.*, 2006), although it should be noted that Mycobacterial EsxA and EsxB interact strongly (Renshaw *et al.*, 2002; 2005) whereas there is no detectable interaction between *S. aureus* EsxA and EsxB (Sundaramoorthy *et al.*, 2008; Anderson *et al.*, 2013). Loss of *esxB* does, however, abolish secretion of EsxC (Fig. 5).

Similar to the *esxB* deletion, the deletion of *esxA* severely affected the secretion of EsxC (Fig. 5). In contrast, deletion of the *esxC* gene did not affect the secretion of EsxA. Thus EsxC depends upon EsxA for secretion but this is not a reciprocal requirement.

From the results presented here we conclude that in *S. aureus* strain RN6390 the Ess secretion system comprises the membrane proteins EsaA, EssA, EssB and EssC, along with the secreted protein EsxA and the cytoplasmic protein EsaB.

### The Ess system in two strains of *S*. *aureus* contributes significantly to virulence in a murine pneumonia model

It has previously been demonstrated that components and secreted substrates of the Ess system in the *S. aureus* Newman strain contribute to virulence in a mouse model of abscess formation (Burts *et al.*, 2008). We therefore first sought to test whether Ess secretion was required for virulence in additional models of infection, and second whether there might be any strain-specific differences in Ess requirement for virulence.

To this end, we constructed complete deletions of the entire 12 gene *ess* loci in each of the RN6390, COL and SA113 strains. Deletion of this locus had no effect on growth rate of the strains in different laboratory growth media (Fig. S4 and data not shown). Loss of *ess* also had no detectable effect on virulence of RN6390 or COL in the insect infection model, *Galleria mellonella* (Fig. S5). We conclude that loss of Ess secretion does not have a phenotypic effect on growth of these *S. aureus* strains.

*Staphylocccus aureus* frequently colonizes the anterior nares of humans, and is an important cause of ventilator-associated pneumonia in hospital settings (e.g. Rocha *et al.*, 2013). It is also one of the earliest bacterial pathogens to colonize the airways of cystic fibrosis patients, where it often persists for many years (e.g. Kahl *et al.*, 1998; Kahl *et al.*, 2003; The United States Cystic Fibrosis Foundation, 2011, Patient Registry Annual Data Report). We therefore sought to assess whether the Ess secretion system was required first for nasal colonization and second for the development of pneumonia in the cystic fibrosis mouse infection model (McCarthy *et al.*, 2010; Twomey *et al.*, 2012).

**Figure 7:**
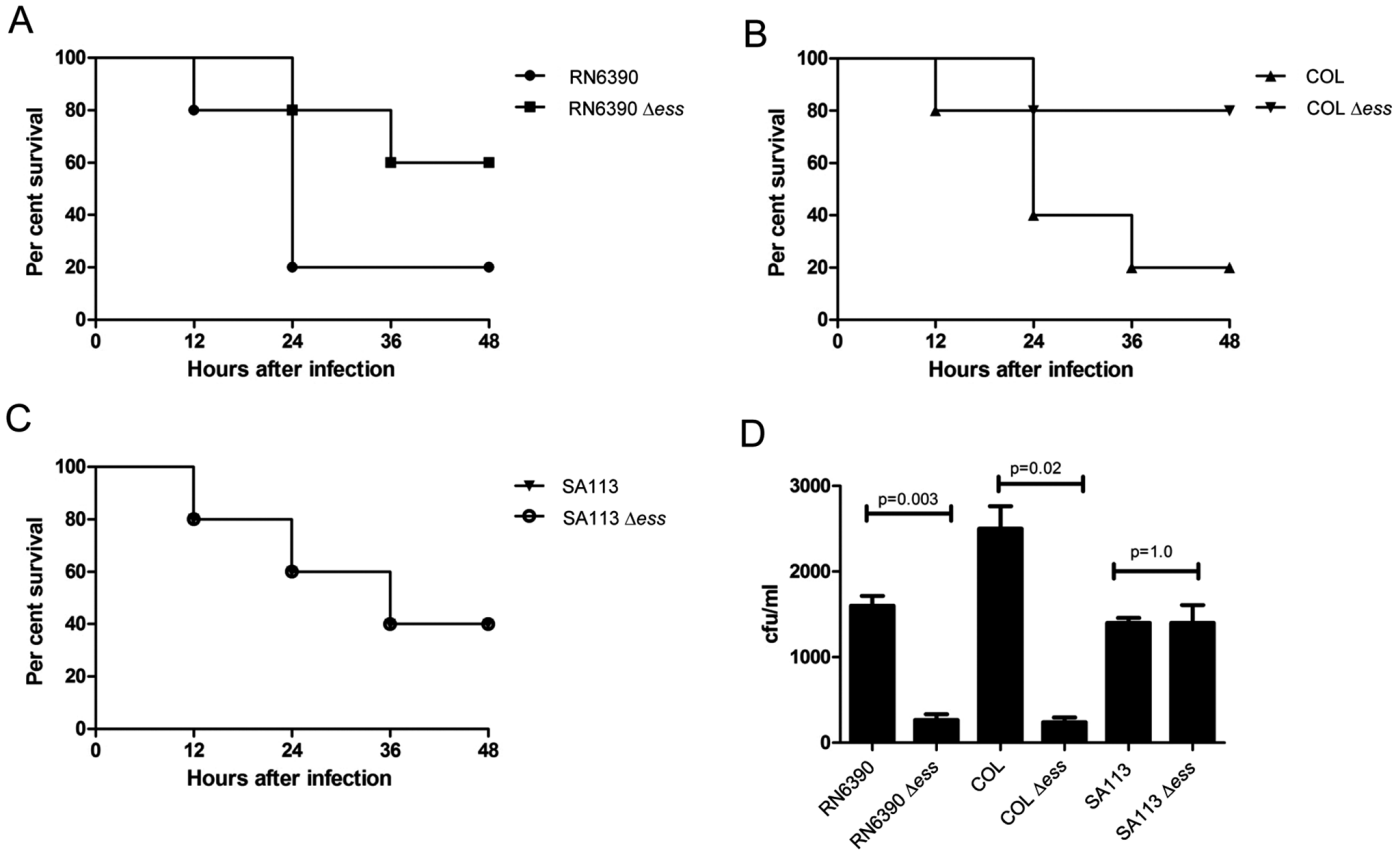
The Ess secretion system of strains RN6390 and COL contributes to nasal colonization and virulence in the murine pneumonia model. For the murine pneumonia model mice (*n* = 10 mice per experiment) were infected at time zero with 2 × 10^8^ cfu of *S*. *aureus* strains RN6390 (A), COL (B) or SA113 (C) or the cognate Δ*ess* mutant strains as indicated. Survival was assessed for up to 48 h. (B) For the murine nasal colonization model, mice (*n* = 10 mice per experiment) were infected with 2 × 10^8^ cfu per nostril of the indicated strains and colonization status determined following nasal excision at 48 h.

To test the ability of RN6390, COL and SA113 or the cognate *ess* deletion strains to colonize the mouse nasal passages, C57BL/6 mice were inoculated with 10^8^ colony-forming units (cfu) of each of the strains, and the number of cfu colonizing the nares were assessed after 3 days. Significantly lower cfu ml^−1^ were recovered from the nares for the Δ*ess* strains of RN6390 (*P* = 0.003) and COL (*P* = 0.02) compared to the cognate wild-type, demonstrating that the Ess system is required to support nasal colonization in these strain backgrounds (Fig. 7D). Surprisingly, no difference in colonizing ability was observed between SA113 and its corresponding Δ*ess* mutant (*P* = 1.0). It should be noted that SA113, which carries a mutation in *agr,* forms more robust biofilms than RN6390 and COL (e.g. Beenken *et al.*, 2003; Herbert *et al.*, 2010), and that SA113 also reportedly produces larger amounts of certain MSCRAMM adhesins, for example Spa, than COL (Herbert *et al.*, 2010). It is possible that these features of SA113 may over-ride for any role of the Ess system in colonization by this strain.

To determine whether the Ess system was required for virulence in the murine pneumonia model, we infected cystic fibrosis transmembrane conductance regulator (CFTR) knockout mice intra-nasally with either of RN6390, COL and SA113 or the cognate *ess* deletion strains (Fig. 7A–C). Again, it can clearly be seen that there were strain-specific differences in the contribution of the Ess secretion system to virulence in the pneumonia model. Thus, mortality was significantly reduced following infection with the Δ*ess* mutants at 48 h in the RN6390 strain background (*P* = 0.03 by log-rank test), and in the COL background (*P* = 0.008). Strain SA113 was less virulent than the other strains we tested in our particular virulence model, which may arise due to the *agr* mutation that is known to lead to a downregulation of virulence factor production (e.g. Abdelnour *et al.*, 1993; Novick *et al.*, 1993). However, again we noted that there no difference in survival was observed between SA113 the cognate Δ*ess* strains (*P* = 1.0).

## Discussion

In this study we have investigated the genetic organization of loci encoding the Type VII/Ess protein secretion system in closely related strains of *S. aureus* and assessed whether the secretion systems are active in these organisms and contribute to virulence. We chose to focus on five strains from clonal complex 8, RN6390, Newman, USA300, SA113 and COL. While the *ess* gene cluster is almost identical in nucleotide sequence between these strains (two nucleotide differences over the approximately 14 kb region), the genetic organization surprisingly differed between RN6390 and Newman on the one hand and USA300, SA113 and COL on the other. In the latter three strains the first gene of the cluster, *esxA*, was coexpressed with *esaA*, which is positioned 83 nucleotides downstream. By contrast, in RN6390, *esxA* was monocistronic and *esaA* was expressed exclusively from a promoter located in the *esxA*-*esaA* intergenic region. Despite the 100% sequence conservation between the five strains over this region, no promoter activity in this intergenic region was detected for USA300.

A putative rho-independent terminator sequence is located in the *esxA*-*esaA* intergenic region. Ostensibly, this must function as a highly effective terminator in RN6390 and Newman, while allowing readthrough in USA300, SA113 and COL. However, despite the surprising differences in transcriptional organization of *esxA* and *esaA* between strains, comparison of transcript levels of these two genes indicate that the ratio of *esxA* and *esaA* transcripts remain remarkably similar. The molecular basis for these unexpected transcriptional differences is not known, but presumably it results from strain-specific variation in the function of *trans*-acting factors. Our additional RT-PCR analyses, coupled with the short intergenic regions between the remaining genes, make it highly likely that *esaA* through *SAOUHSC_00269* are all coexpressed.

In addition to the heterogeneity of *ess* transcriptional organization, we also saw that there were notable differences between strains in the activity of the Ess secretion system. During growth in TSB medium strains RN6390 and COL secreted EsxA and EsxC at relatively early growth points, whereas for USA300 and SA113, secretion appeared to be delayed and was only observed at later growth points. We were unable to observe any secretion of EsxA in strain Newman at any of the growth points we tested, and could not detect EsxC antigen. It has been noted previously that Newman produces and secretes EsxA at a low level relative to USA300 (Burts *et al.*, 2008). Recently, it was shown that the *ess* cluster in Newman and USA300 is negatively regulated by the SaeRS two-component system, but that this system is constitutively active in the Newman strain due to a point mutation in the SaeS kinase, accounting for the low Ess activity (Anderson *et al.*, 2013).

Selecting one of the early secreting strains, RN6390, we made a series of in-frame deletions in the first eight *ess*-encoded genes to determine whether they were essential to support Ess secretion system activity. Our results were in partial agreement with prior observations in strain Newman (Burts *et al.*, 2005; 2008,) – thus we confirmed in the RN6390 background that the EssA, EssB and EssC membrane components are required for secretion of EsxA and EsxC. However, we also demonstrated an essential role for a further membrane protein, EsaA, in secretion system activity. This contrasts with Burts *et al.* (2005) who reported that a transposon insertion in *esaA* did not abolish secretion of EsxA or EsxB. However in that study the transposon was inserted after codon 370 of *esaA*, raising the possibility that a truncated protein product was produced that may retain some function. It should be noted that EsaA homologues are conserved in *Firmicutes* and the *B. subtilis* EsaA homologue, YueB, is essential for Ess secretion activity in this organism (Baptista *et al.*, 2013). Taken together, these results strongly suggest that EsaA proteins are essential components of the Ess secretion machinery.

A striking finding was that for all strains examined, secretion appeared to be relatively inefficient, with significant amounts of EsxA and EsxC detected in the cellular fraction, and subcellular fractionation of RN6390 showed that they were primarily located in the cytoplasm. We also noted that for USA300 and SA113, cellular EsxA was detected at early growth points, but did not appear in the growth medium until later. These findings raise the possibility that the system may be subject to post-translational regulation. Other protein secretion systems are also regulated at the post-translational level, for example the Type VI secretion system in Gram-negative bacteria. In *Pseudomonas aeruginosa* it has been shown that a serine-threonine kinase, PpkA, phosphorylates a FHA domain-containing protein, regulating the activity of the Type VI secretion system (Mougous *et al.*, 2007). It is interesting to note that in *Firmicutes*, the EssC component has tandem FHA domains at its N-terminus (Tanaka *et al.*, 2007) that may be involved in controlling secretion activity. The small ubiquitin-related protein, EsaB, could also play a role in post-translational regulation of the secretion system. Here we found that *esaB* is essential for secretion of EsxA and EsxC, and Baptista *et al.* (2013) similarly reported that the *B. subtilis* EsaB homologue, YukD, was required for activity. By contrast, in the *S. aureus* Newman strain EsaB is dispensable for secretion of EsxA or EsxB but has a negative regulatory effect on the expression of *esxC* at the post-transcriptional level (Burts *et al.*, 2008). Given that EsaB is not universally essential for Ess secretion it is unlikely to be a structural component of the machinery. Instead we favour the idea that it plays a regulatory role albeit one that may differ between strains.

Our qRT-PCR analysis has shown that *esxA* is very highly expressed relative to the other genes at the *ess* locus, approximately 100 times more than *essC* or *esxB*. The role of EsxA in Type VII secretion systems is not completely clear. It is evident that it is secreted by the Type VII machinery, but deletion or insertional activation of *esxA* always results in a complete absence of all other secreted substrate proteins in the growth medium (Burts *et al.*, 2005; Anderson *et al.*, 2013; this study). In this respect it behaves like one of the core components of the secretion machinery (potentially an extracellular structural component or chaperone) rather than a secreted substrate. By contrast, deletion of genes coding for two further secreted substrates, *esxB* and *esxC*, did not affect secretion of EsxA in RN6390, placing EsxA at the top of the hierarchy of Ess-secreted proteins. It should be noted that complex effects have been observed in other strain backgrounds, for example EsxA was destabilized in the *esxB* mutant of Newman (Burts *et al.*, 2005) but not USA300, although EsxA was not secreted in this case (Anderson *et al.*, 2013). Likewise, absence of EsxC, with which EsxA was demonstrated to interact, appeared to destabilize EsxA in USA300 (Anderson *et al.*, 2013), but this was not evidenced here for RN6390. Such complex, strain-specific effects make it difficult to tease out the roles of individual secreted proteins in secretion system function and to pin-point what contribution, if any, each secreted protein has to *S. aureus* virulence.

Prior reports have defined a role for the Ess system of strain Newman in virulence in a murine abscess infection model. Here we asked whether the Ess system was required for *S. aureus* to colonize the murine nasal passage, which is usually asymptomatic and benign, and to cause pneumonia following intra-nasal inoculation. Surprisingly we found for two of the three strains tested, RN6390 and COL, that the Ess system was important for both of these processes. Currently it is not clear how the Ess system could contribute to nasal colonization, although it has been observed that other secretion systems also play a major role in this bacterial behaviour (e.g. Skinner *et al.*, 2004). Very recently it was reported that the *essC* gene in strain Newman was upregulated in the presence of pulmonary surfactant and that deletion of *essC* was accompanied by a similar attenuation in virulence in the murine pneumonia model as we report here (Ishii *et al.*, 2014). By contrast, the third strain we tested, SA113, showed no significant contribution of the Ess system to either colonization or disease. The underlying reasons for these strain-specific differences are not known, and do not entirely correlate with the activity of the Ess secretion systems in laboratory growth media. Taken together with previous findings, the results presented here demonstrate that there are notable differences between the transcriptional organization of the *ess* locus, the absolute requirement of individual *ess*-encoded genes for secretion system function, secretion activity in laboratory broth and requirement for virulence between relatively closely related strains of *S. aureus* and that findings cannot necessarily be extrapolated between strains. Therefore, the precise role of individual machinery components and contribution of specific secreted substrates to disease requires further study.

## Experimental procedures

### Bacterial strains and growth conditions

Strains and plasmids used in this study are listed in Table 1. *S. aureus* strains were grown in TSB at 37°C under vigorous agitation. Chloramphenicol (Cm) at a final concentration of 10 μg ml^−1^ or erythromycin (Erm, 5 μg ml^−1^) was added for plasmid selection. Anhydrotetracycline (ATc) was used at various concentrations as a selection during allelic gene replacement using the pIMAY system [1 μg ml^−1^ (Monk *et al.*, 2012)] or for induction of target gene expression from the pRMC2 plasmid (Corrigan and Foster, 2009). *Escherichia coli* was grown aerobically in Luria–Bertani (LB) medium at 37°C. If required, cultures were supplemented with ampicillin (Amp, 100 μg ml^−1^) or chloramphenicol (15 μg ml^−1^) for plasmid selection.

**Table 1:** Strains and plasmids used in this study

Strain or plasmid	Description	Reference
Strains		
*S. aureus*		
RN6390	NCTC8325 derivative, *rbsU*, *tcaR*, cured of φ11, φ12, φ13	Novick *et al.* (1993)
COL	MRSA, *agr*	Dyke *et al.* (1966), Gill *et al.* (2005)
USA300	Community-acquired MRSA	McDougal *et al.* (2003)
Newman	ATCC 25904	Duthie and Lorenz (1952), Baba *et al.* (2008)
SA113	NCTC8325 derivative, *agr*, *rbsU*, *tcaR, hsdR*	Iordanescu and Surdeanu (1976)
RN6390 ess	Complete deletion from *esxA* – *SAOUHSC_00269*	This work
RN6390 esxA	*esxA* deletion	This work
RN6390 esaA	*esaA* deletion	This work
RN6390 essA	*essA* deletion	This work
RN6390 esaB	*esaB* deletion	This work
RN6390 essB	*essB* deletion	This work
RN6390 essC	*essC* deletion	This work
RN6390 esxC	*esxC* deletion	This work
RN6390 esxB	*esxB* deletion	This work
COL ess	Complete deletion from *Sacol0271* (*esxA*) -*Sacol0282*	This work
SA113 ess	Complete deletion from *esxA* – *SAOUHSC_00269*	This work
*E. coli*		
JM110	*rpsL thr leu thi lacY galK galT ara tonA tsx dam dcm glnV44* Δ(*lac-proAB*) e14-[F' *traD36 proAB*^+^ *lacI*^q^ *lacZ*ΔM15] *hsdR17*(r_K_^−^m_K_^+^)	Stratagene
MC1061	F^−^ Δ(*ara-leu*)7697 [*araD139*]_B/r_ Δ(*codB-lacI*)3 *galK16 galE15* λ^−^ *e14*^−^ *mcrA0 relA1 rpsL150*(strR) *spoT1 mcrB1 hsdR2*(r^−^m^+^)	Casadaban and Cohen (1980)
DC10B	*dam^+^ dcm^−^ hsdRMS endA1 recA1*	Monk *et al.* (2012)
Plasmids		
pBluescript KS^+^	General purpose *E. coli* cloning vector, amp^r^	Stratagene
pMAD	*E. coli/S. aureus* shuttle vector, temperature sensitive, amp^r^, ery^r^	Arnaud *et al.* (2004)
pIMAY	*E. coli/S. aureus* shuttle vector, temperature sensitive, cml^r^	Monk *et al.* (2012)
pRMC2	*E. coli/S. aureus* shuttle vector, inducible protein expression, amp^r^, cml^r^	Corrigan and Foster (2009)
pRMC2h	pRMC2 variant coding for N-terminal hexahistidine tag	This work
pEsaA-nhis	pRMC2h expressing His_6_-EsaA	This work

### Strain and plasmid construction

In-frame deletions of individual *ess* genes or a 12-gene *ess* deletion (encompassing *esxA* through *SAOUHSC_00269*) were performed by allelic exchange using either plasmid pMAD (for *essA*, *esaB*, *essB*, *esxC*, *esxB*) or pIMAY (for *esxA*, *esaA*, *essC*, and the 12 gene *ess* deletion) as described (Arnaud *et al.*, 2004; Monk *et al.*, 2012). For each gene, the upstream and downstream regions including at least the first three and last three codons were amplified from RN6390 genomic DNA using primers listed in Table S1. In the case of overlapping genes within the *ess* cluster (*esaA-essA*, *essA-esaB*, *esxB-esxD*), the amplified flanking regions were designed to leave the putative ribosome binding site and start codon or stop codon of the overlapping gene intact. Clones containing the amplified flanking regions in vectors pMAD or pIMAY were selected in *E. coli* and plasmids were subsequently electroporated into *S. aureus* strains using published methods (Monk *et al.*, 2012). Chromosomal deletions were verified by amplification of the genomic region from isolated genomic DNA (GeneElute Bacterial Genomic DNA Kit, Sigma Aldrich) and DNA sequencing of the amplified products. To ensure that no unwanted secondary mutations arose in virulence regulators such as *agr* or *sae*, each mutant was also tested for haemolytic activity on sheep blood agar plates. In each case no detectable difference in haemolytic activity between the wild-type and any of the mutant strains was observed.

A variant of plasmid pRMC2 coding for an N-terminal hexahistidine tag (pRMC2h) was generated by insertion of the additional coding sequence into the multiple cloning site (MCS). Briefly, the region between the XhoI site and the MCS of pRMC2 was amplified with primers pRMC2seq1 and Nhisins, adding an 18 bp stretch corresponding to the short sequence of DNA from the ribosome binding site to the ATG start codon of the *esxA* gene followed by the hexahistidine coding sequence (6× CAT repeats) between the KpnI and BglII restriction sites of the MCS. pEsaA-nhis was constructed by amplification of the *esaA* coding region from RN6390 genomic DNA using primers esaA nhis fw and esaA nhis rev (see Table S1) and cloning between the BglII and SacI sites of pRMC2h.

### RT-PCR and 5′-RACE experiments

mRNA extraction from *S. aureus* strains grown aerobically in TSB to an OD_600_ of 1 or 2 was performed using the SV Total RNA Isolation Kit (Promega) with some minor modifications. Briefly, cell samples were stabilized in 5% phenol/95% ethanol and centrifuged at 2770 *g* for 10 min. Cells were resuspended in 100 μl TE buffer containing 500 μg ml^−1^ lysostaphin and 50 μg ml^−1^ lysozyme and incubated at 37°C for 30 min followed by isolation of the RNA according to the manufacturer's instructions. Isolated RNA was further purified using the DNA-free kit (Ambion) to remove DNA and salt.

RT-PCR to probe co-transcription of *ess* genes was carried out using the Superscript III Reverse Transcriptase kit (Invitrogen) using region-specific primers (region-x-f and region-x-r) listed in Table S1, followed by incubation with 2 units of *E. coli* RNaseH (Invitrogen) to remove template RNA. PCR products were purified using the PCR purification kit (Qiagen) and visualized on 1% agarose gels.

First-strand cDNA synthesis for 5′-RACE was performed following the protocol for the Roche second-generation 5′/3′-RACE kit using 500 ng of template RNA prepared as described above, gene-specific primers for *esxA* or *esaA* GSP1-3 and anchor primers listed in Table S1. Single PCR products were purified using the PCR purification kit (Qiagen), digested with ClaI/XhoI (esxA) or ClaI/EcoRI (*esaA*) and cloned into pBluescript KS^+^. Transcriptional start points were determined by sequencing with primer M13-F.

### RT-qPCR

RNA was extracted as described above and 600 ng of total RNA was used to generate 20 μl of cDNA using the QuantiTect Reverse Transcription Kit (Qiagen), following the manufacturer's instructions. For each sample, a negative control was prepared replacing the enzyme mixture with additional water. Quantitative PCR was performed using a Stratagene Mx3005P thermal cycler. Triplicate reactions for each culture condition were set using 7.5 μl Brilliant II SYBR Green Low Rox SuperMix (Agilent), 0.6 μl 10 mM primers (see Table S1) and 3.0 μl of cDNA diluted 1:20, and made up to 15 μl with sterile water. PCR was performed using an initial 10 min 95°C denaturing step followed by 40 repeated cycles of 30 s 95°C denaturing, 30 s 55°C annealing, and 30 s 72°C extension steps. A final 10 min denaturing curve analysis was performed. Standard curves were generated from serial 10-fold dilutions of genomic DNA (see Fig. S1). Amplification results were analysed with MxPro QPCR software to give the levels of mRNA normalized to the level of 16S rRNA amplification in each sample. Results were further analysed in Microsoft Excel to calculate relative expression levels using *essC* mRNA as the comparator.

### Antibody production

Coding sequences for *S. aureus* EsxA, EsaC (Uniprot: Q99WU4 and Q99WT8), predicted cytoplasmic fragments of EssB (Uniprot: Q99WU0, residues: 12–226) and EssC (Uniprot: Q932J9, residues: 964–1479) and predicted extracellular fragment of EsaA (Uniprot: Q99WU3, residues: 313–749) were PCR amplified from synthetic genes [codon optimized for *E. coli* K12 (Genscript)] and individually cloned between the SalI/XhoI sites of a modified pET27b vector (Novagen). All primers are listed in Table S1. The plasmids produce proteins with an N-terminal hexahistidine tag separated by a tobacco etch virus (TEV) protease cleavage site. Proteins were expressed and purified as reported previously (Zoltner *et al.*, 2013a), except in the final size exclusion chromatography step a HR 30/100 GL Superdex75 column (CV = 24 ml, GE healthcare) equilibrated with 20 mM Tris pH 7.8, 100 mM NaCl was used.

The purified proteins (retaining a Gly–Ala–Ser–Thr sequence at the N-terminus after the cleavage step) were utilized as antigens to immunize rabbits for polyclonal antibody production in a standard three injections protocol (Seqlab, Goettingen, Germany).

The anti-TrxA antibody was described earlier (Miller *et al.*, 2010) and commercially available antibodies against Spa (HRP-conjugate, LifeSpan Biotechnologies) and SrtA (Abcam) were used according to the manufacturers' recommendations.

### *S*. *aureus* sample preparation and Western blotting

*Staphylococcus aureus* strains were grown overnight in TSB at 37°C. Cells were diluted 1/100 into fresh TSB medium and growth was monitored by measuring the optical density of the cultures at 600 nm. At indicated time points, samples were withdrawn and cells pelleted by centrifugation at 2770 *g*. Supernatant samples were further filtered through a 0.45 μm filter and proteins precipitated with trichloroacetic acid (TCA, 10% final concentration) in the presence of 50 μg ml^−1^ deoxycholate, which has been reported to improve recovery of precipitated proteins (Bensadoun and Weinstein, 1976). Precipitates were centrifuged (10 000 *g*, 4°C, 15 min), washed with 80% ice-cold ethanol and resulting pellets resuspended in 50 mM Tris-HCl pH 8, 4% SDS. Samples were mixed with NuPage LDS loading buffer (life Technologies) and boiled for 10 min.

Cells for whole cell fractions were pelleted by centrifugation, washed with 1× PBS buffer and normalized to an OD_600_ of 2 in 1× PBS. Cells were lysed by addition of 50 μg ml^−1^ lysostaphin (Ambi) and incubation at 37°C for 30 min. Samples were mixed with an equal volume of LDS buffer and boiled for 10 min.

Samples for further subcellular fractionation were pelleted and washed as above and resuspended in fractionation buffer (50 mM Tris-HCl pH 7.6, 0.5 M sucrose, 10 mM MgCl_2_). Lysostaphin was added as above and the cell wall was digested at 37°C for 30 min. Protoplasts were sedimented (10 000 *g*, 10 min) and the supernatant kept as the cell wall fraction. Protoplasts were resuspended in 1× PBS and broken by sonication (2 × 15s, 20% amplitude, Branson Digital Sonifier). Samples were subjected to ultracentrifugation (227 000 *g*, 30 min, 4°C), the supernatant was removed as the cytoplasmic fraction and the membrane pellet resuspended in 1× PBS, 0.5% Triton X-100.

Samples were mixed with LDS loading buffer and boiled for 10 min prior to separation on bis-Tris gels. Western blotting was performed according to standard protocols with the following antibody dilutions: α-EsxA 1:2500, α-EsxC 1:2000, α-EsaA 1:10 000, α-EssB 1:10 000, α-EssC 1:10 000, α-TrxA 1:25 000, α-Hla 1:2000, α-Spa 1:10 000, α-SrtA 1:3000.

### Virulence assays

For the *G. mellonella* virulence assay, overnight cultures of *S. aureus* and the isogenic Δ*ess* mutant were used to prepare a 10-fold dilution series in phosphate-buffered saline (PBS). The infecting dose of wild-type RN6390 and COL strains required to cause 80% mortality (LD_80_) in the model was determined (1 × 10^7^ cfu ml^−1^ for RN6390 and 1 × 10^8^ cfu ml^−1^ for COL) and used in subsequent experiments. Larvae were removed from storage at 4°C and allowed to warm to room temperature. Prior to inoculation, larvae were briefly placed on ice; then a Hamilton syringe was used to inject larvae with 10 μl of culture in PBS via the hind left proleg (*n* = 10 larvae per experiment; repeated three times). Ten control larvae were injected with PBS only. Following injection, larvae were incubated in the dark at 25°C. After 24 h, larvae were scored as dead or alive, with larvae considered dead if they did not respond to touch and were usually visibly black. Infection models were continued for 7 days and differences in survival assessed using the Kaplan-Meier method.

The murine pneumonia model was performed using CFTR −/− mice, a relevant model of pulmonary *S. aureus* infection (Bragonzi, 2010) as *S. aureus* is the most common organism colonizing children with cystic fibrosis. Briefly, *S. aureus* strains were grown in TSB medium at 37°C overnight with shaking, after which bacteria were collected by centrifugation and resuspended in PBS. The exact number of bacteria was determined by plating serial dilutions of each inoculum on Luria broth agar plates. Female C57BL/6 mice CF transmembrane conductance regulator (CFTR) knockout (CF) mice (approximately 10 weeks old) were anaesthetized and infected by the intranasal route with 10 μl of culture of RN6390, RN6390 Δ*ess*, COL, COL Δ*ess*, SA113 and SA113 Δ*ess* strains to have a final inoculum of 2 × 10^8^ colony-forming units (cfu) per mouse. Survival was assessed up to 48 h after induction of pneumonia and compared using the Kaplan-Meier method.

Murine nasal colonization assays were carried out as previously reported (Kiser *et al.*, 1999). Briefly, an inoculum of each of the *S. aureus* strains, 10^8^ cfu in 10 μl of PBS, was pipetted slowly onto the nares of wild-type female C57BL/6 mice. After inoculation, mice were killed 3 days post-infection by intraperitoneal injection of 0.3 ml of 30% pentobarbital. Nares were harvested aseptically and homogenized in sterile PBS. A 10-fold serial dilution and plating was carried out to assess cfu. All animal experiments were approved by the Animal Ethics Committees of University College Cork.
