# Supplementary material for: Heterogeneity in ess transcriptional organization and variable contribution of the Ess/Type VII protein secretion system to virulence across closely related Staphylocccus aureus strains
Source: Mol Microbiol. 2014 Jul 30;93(5):928–43. doi: 10.1111/mmi.12707 (PMC4285178; doi:10.1111/mmi.12707)
Supplement: Supplementary file 1 — Supporting information [file mmi0093-0928-SD1.pdf]

Supplementary Figures:

Fig S1. Relative standard curves for RT qPCR analysis of selected genes at the *ess* locus. RT-qPCR standard curves for: (A) 16S rRNA; (B) *esxA*; (C) *esaA*; (D) essC and (E) *esxB*. Standard curves were prepared as described in the materials and methods section using serial 10-fold dilutions of RN6390 genomic DNA. Data points represent individual results from three technical repeats at each dilution. Regression curves to calculate the efficiency of each primer pair were calculated using MxPro software (Stratagene).

Fig S2. EsaA, EssB and EssC presence in RN6390 and individual *ess* deletion strains.

The RN6390 wild-type (wt) or isogenic deletion strains, as indicated, were cultured in TSB medium until an OD_{600} of 2 was reached. Whole cell samples were prepared as described in the Methods section, adjusted to a calculated cell density of OD 1 in LDS sample buffer and separated on bis-Tris gels (8 % for EsaA and EssC blots, 10% for EssB and 15% for TrxA). Immunoblotting was undertaken with anti-EsaA, anti-EssB, anti-EssC or anti-TrxA antibodies. An equivalent of 10 µl of cells from an OD_{600} of 1 was loaded for each lane. Molecular weight markers are indicated to the left of each blot, and the specific protein band recognised by each antibody is indicated by an arrow to the right. The asterisk indicates a non-specific cross-reacting band.

Fig S3. EsxA and EsxC secretion are restored to the *esaA* mutant strain by *in trans* expression of a his-tagged allele of *esaA*.

The RN6390 wild-type strain (wt), or the isogenic *esaA* mutant strain containing either pRMC2 empty vector (*esaA*), or pRMC2 encoding an N-terminally hexahistidine-tagged EsaA variant (*esaA* p*esaA*) were cultured in TSB medium. When an OD_{600} of 0.5 was reached, one culture of *esaA* p*esaA* was supplemented with 100 ng/ml anhydrotetracycline

to induce plasmid-encoded overexpression of his-tagged EsaA (+++). All strains were subsequently grown until they reached OD_{600} of 2. The cells were subsequently spun down and the supernatant (sn) was retained as the secreted protein fraction, while the pellet was retained as the cellular fraction. (A) Samples of the supernatant and cellular fractions (An equivalent of 250 μl of supernatant and 10 μl of cells adjusted to OD1) were separated on bis-Tris gels and immunoblotted using the anti-EsxA or EsxC antisera, or control antisera raised to TrxA (cytoplasmic protein). (B) Samples of the cellular fractions were separated on bis-Tris gels and immunoblotted using the anti-EsaA polyclonal antisera. The asterisk indicates a non-specific cross-reacting band.

Fig S4. Deletion of the 12 gene *ess* locus has no effect on *S. aureus* RN6390 growth. The RN6390 wild-type (wt) or complete *ess* deletion (Δ*ess*) strains were inoculated into 100 μl volumes of either complex (TSB) or defined (RPMI) growth media in 96 well plates and cultured aerobically at 37°C for the indicated time period. Note that in this growth format, optical density at 600nm did not exceed 1 unit, whereas in batch culture it routinely reaches OD_{600} >6.

Fig S5. The ESS system is dispensable for *S. aureus* virulence in the wax moth larvae infection model. Kaplan-Meier survival curves showing the survival of *Galleria Mellonella* larvae over seven days following infection with (A) *S. aureus* strain RN6390 and the isogenic *ess* deletion (Δ*ess*) strain and (B) *S. aureus* strain COL and the isogenic *ess* deletion (Δ*ess*) strain. Curve comparisons are not statistically significant using the log rank test (p=0.6 for RN6390 and p=0.5 for COL). Curves are generated from n=30 larvae in each group (experiments performed as 10 in each group repeated 3 times). Larvae injected with 10µl of phosphate-buffered saline as a control (not shown) demonstrated no mortality over 1 week of observation (n=10).

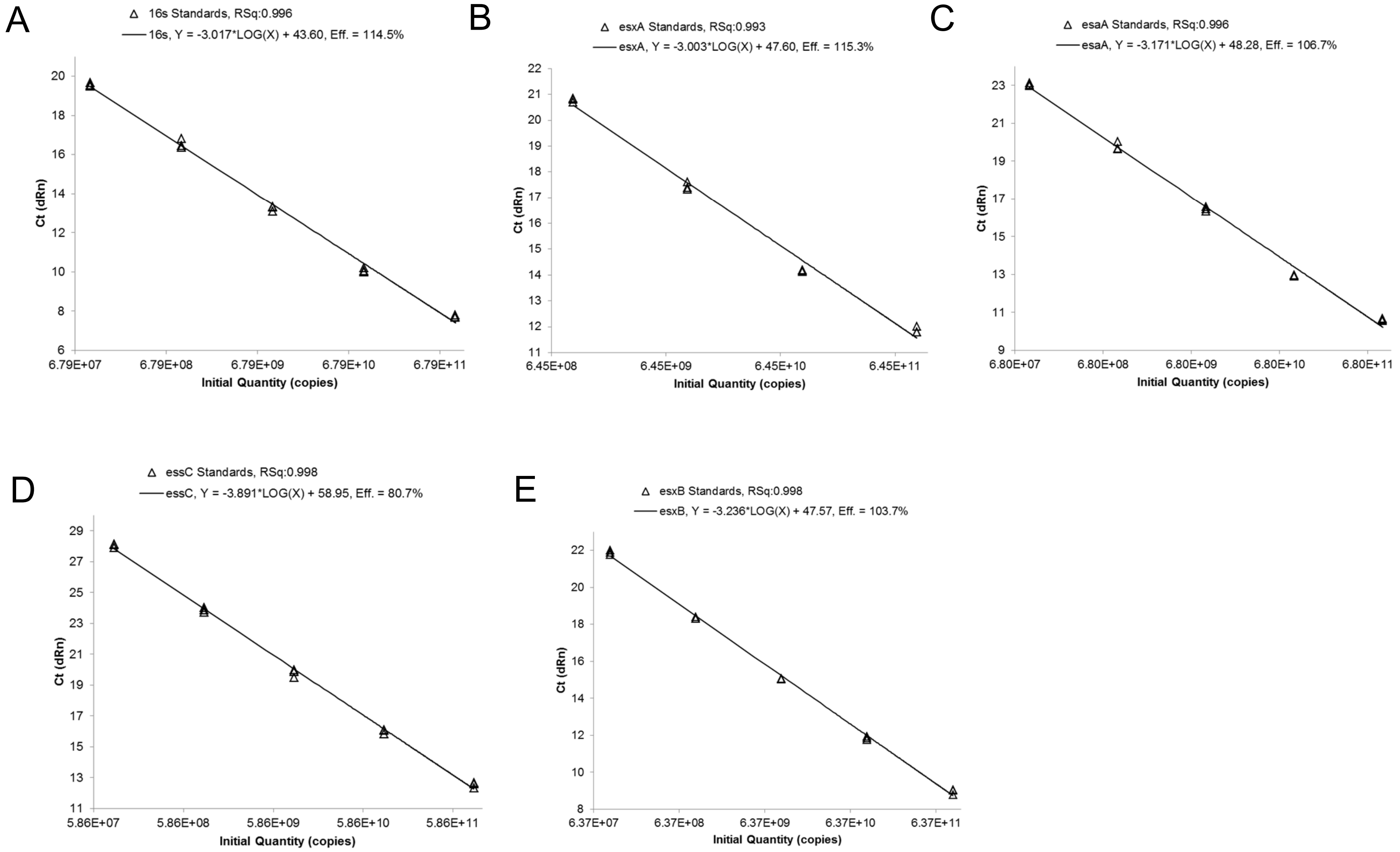

Kneuper *et al*. Fig S1

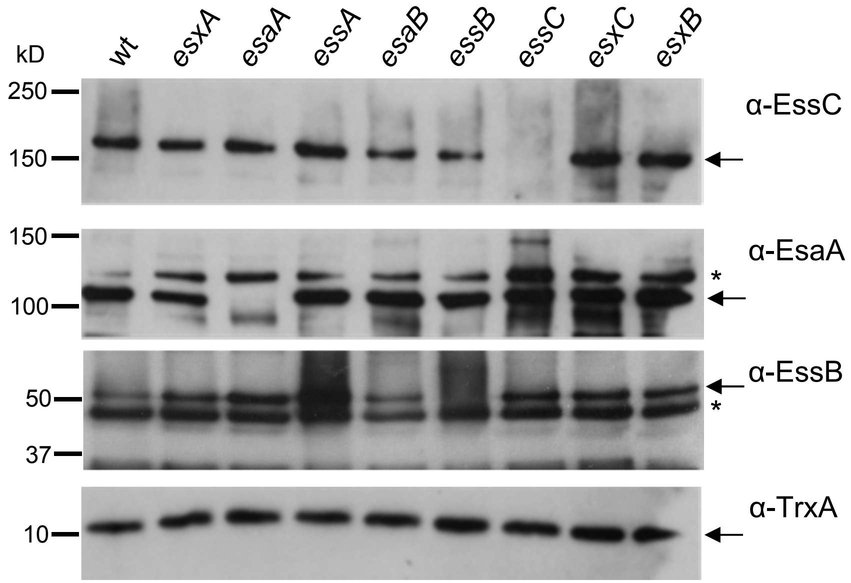

Kneuper *et al*. Fig S2

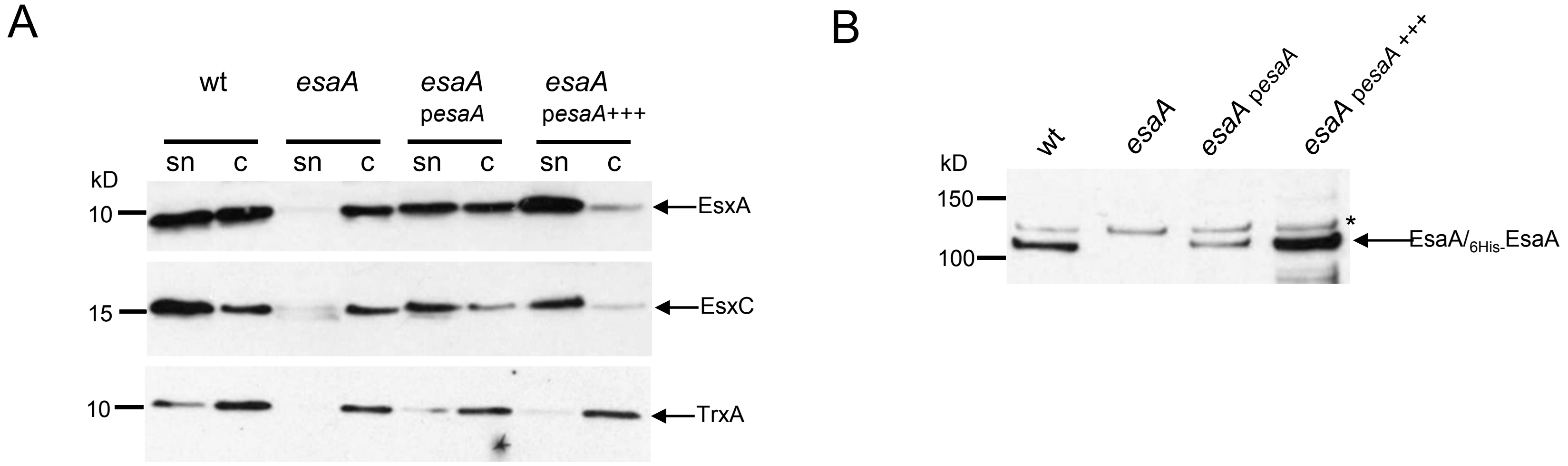

Kneuper *et al*. Fig S3

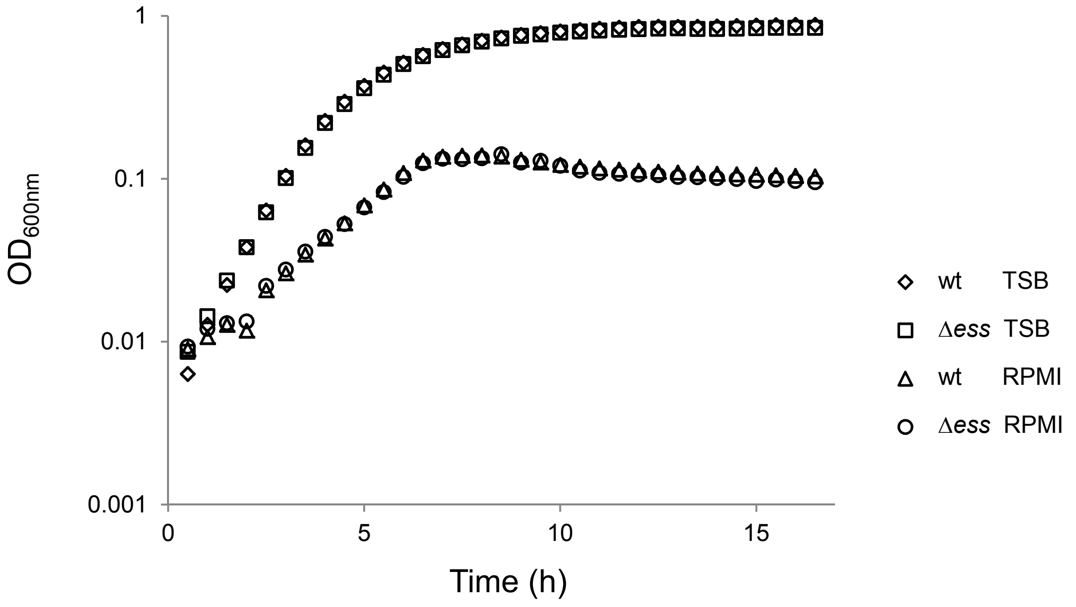

Kneuper *et al*. Fig S4.

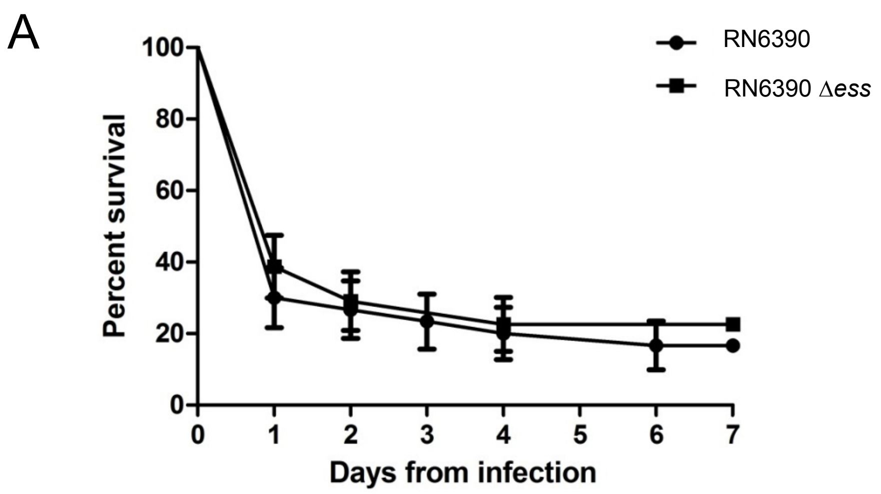

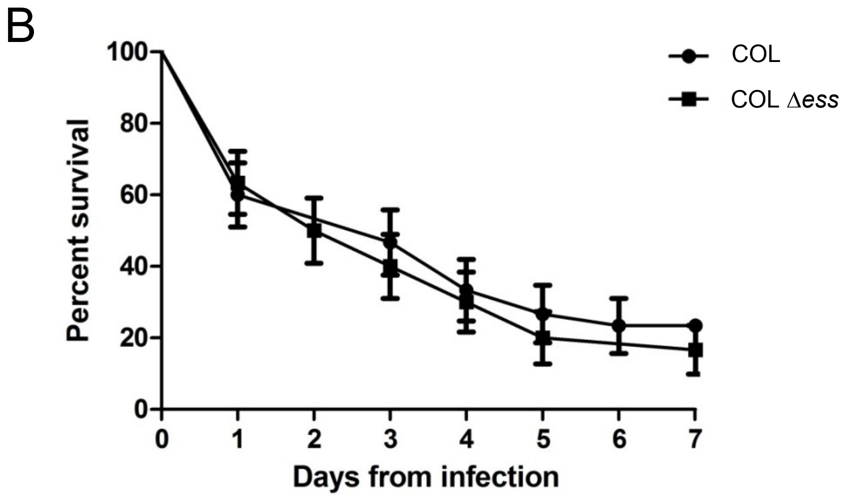

Kneuper *et al*. Fig S5.

Primer	Sequence (5'-3')	Usage
EsxAdA1	AATGTGCGAATTCTGACCAC	Amplification of
EsxAdA2	TGCTTATTGCATTGCCATAACTAGAAACC	*esxA* flanking
EsxAdB1	TTGTTGAATTCGTTAAGTAC	regions for cloning
EsxAdB2	ATGGCAATGCAATAAGCATTCTGAAATTG	into pIMAY
EsaAdA1	TAGGATCCACATTTATATTACAAAAATG	Amplification of
EsaAdA2	AAAGTGTTTCTTTTTCATGCTTATTTCC	*esaA* flanking
EsaAdB1	ATTGAATTCTCCAAACTATCC	regions for cloning
EsaAdB2	ATGAAAAAGAAACACTTTAAGAAAGAGAG	into pIMAY
EssAdA1	TGATGAATTCACTATCAATG	Amplification of
EssAdA2	AGTCAAAAACATCAACATTAGATTAATCTC	*essA* flanking
EssAdB1	GTCATAATGAATTCGGAAAC	regions for cloning
EssAdB2	ATGTTGATGTTTTTGACTTTAGGATTTGTC	into pMAD
EsaBdA1	ACTAATAGAATTCCACCATTG	Amplification of
EsaBdA2	CTATAGTAAATCAAATGTTACTTTTACGTG	*esaB* flanking
EsaBdB1	AAGGATCCTTCAACTAAAGCATCAAATG	regions for cloning
EsaBdB2	ACATTTGATTTACTATAGGAGGAAAAATAG	into pMAD
EssBdA1	AATGATGCGAATTCTAAGCAG	Amplification of
EssBdA2	CTATTTTTTTTTAACCATCTATTTTTCCTC	*essB* flanking
EssBdB1	ATGCCACTGAATTCTGACTC	regions for cloning
EssBdB2	ATGGTTAAAAAAAAATAGTATAGGACTGAG	into pMAD
EssCdA1	AGTACTGAATTCGTATGATG	Amplification of
EssCdA2	AAACCATCTTTTATGCATTGTCTTTGCCTC	*essC* flanking
EssCdB1	AGGGATCCGTTGCGTTTGCTTTTGACATG	regions for cloning
EssCdB2	ATGCATAAAAGATGGTTTAAATAGCAATG	into pIMAY
EsxCdA1	AACGGATCCAGCAATGATTTCATCAG	Amplification of
EsxCdA2	TTAATTCATAAAATTCATAACATACCTCC	*esxC* flanking
EsxCdB1	TCATGAATTCGAATATTTACAATGGCGC	regions for cloning
EsxCdB2	ATGAATTTTATGAATTAATATTGAGGTGAAG	into pMAD
EsxBdA1	AGGAATTCGTATTTCAGACCAACAATTC	Amplification of
EsxBdA2	CACCCTATCTCCACCCATATCTTCACCTC	*esxB* flanking
EsxBdB1	TCGGATCCAAATAATTCTCGTATAAATG	regions for cloning
EsxBdB2	ATGGGTGGAGATAGGGTGAACCCATGATG	into pMAD
EsxAdA1	AATGTGCGAATTCTGACCAC	Amplification of *ess*
EsxdA2	TTATTCTTCCATTGCCATAACTAGAAACC	operon flanking
EsxdB1	GAAGAGCTCATAATGATTTTGTACAGC	regions for cloning
EsxdB2	ATGGCAATGGAAGAATAAACTATCTTAATG	into pIMAY
pRMC2seq1	ATTTGGATCCCCTCGAGTTCATG	His-tag insertion
Nhisins	AAAGATCTTCCTGAATGATGATGATGATGATGC<br>ATAACTAGAAACCTCCTGGTACCGC	N-terminal his-tag<br>insertion pRMC2
esaA nhis fw	GGAAGATCTAAAAAGAAAAATTGGATTTATG	*esaA* cloning into
esaA nhis rev	GGTGAGCTCATTAGATTAATCTCTCTTTCTTAAA<br>G	pRMC2h
pET27bmodEsxA	GCGCGTCGACAATGGCGATG	Antibody production
	GCGCCTCGAGTTACTGCAGGCCAAAGTTGTTG<br>C	Antibody production
pET27bmodEsaA	GCGCGTCGACACAGACCGTGAAAGAAAACCTG	Antibody production

	GATAAAC	
	GCGCCTCGAGTTACGCGTGCAGTTTGTTCACGTTGTTATC	Antibody production
pET27bmodEsaC	GCGCGTCGACAATGAACTTTAACGATATTGAAACG	Antibody production
	GCGCCTCGAGTTAGTTCATCGCTTTGTTAAAATATTCGCTCG	Antibody production
pET27bmodEssB	GCGCGTCGACACAGGATATGCTGACCCCGCTGGATG	Antibody production
	GCGCCTCGAGCTACACGGTATGGCCCACTTTGCGCAC	Antibody production
pET27bmodEssC	GCGCGTCGACGAAGATCTGGTGGAAACCG	Antibody production
	GCGCCTCGAGTTATTTAAACCAGCGAATTTTCTG	Antibody production
region-1-f	CAGGAGGTTTCTAGTTATGGC	RT-PCR
region-1-r	GTTCTTGAACGGCATCAGC	RT-PCR
region-2-f	TTACGGGCAAGGTTCAGACC	RT-PCR
region-2-r	GTAAATAATTCCGGGAAGTCG	RT-PCR
region-3-f	CTAAGACAGGTAAAATCTATCGG	RT-PCR
region-3-r	TGCTTCTTCAGCATCTCTAAAGGCG	RT-PCR
region-4-f	GCATATGTACGCAAAGTAGGAC	RT-PCR
region-4-r	TCGTTAGTTGCTCTTGAGTTC	RT-PCR
region-5-f	CTTGAACATTTTATTTGTCGGC	RT-PCR
region-5-r	AGCCACTTTAAAACCTGCATC	RT-PCR
region-6-f	ATGCAGGTTTTAAAGTGGCTAC	RT-PCR
region-6-r	CGCATCATCCATTGTTGTATCT	RT-PCR
region-7-f	TGGGTCAAAACATAAAGCGTGC	RT-PCR
region-7-r	TCGCATGATGTCCATGGTTC	RT-PCR
esxA-GSP1	GTTCTTGAACGGCATCAGC	5' RACE
esxA-GSP2	ACGGCATCAGCAGTGCTATTC	5' RACE
esxA-GSP3	CGCGCTCGAGATTTCTTCTAATAATTGTGC	5' RACE
esaA-GSP1	TCAAACCAGACTCAGCAAGG	5' RACE
esaA-GSP2	AATGCTTGACCCAGCTCAAC	5' RACE
esaA-GSP3	CGCGAATTCCCTTTTTCTGATTGATCTCC	5' RACE
Anchor primer1	GACCACGCGTATCGATGTCGACTTTTTTTTTTTTTTTTV	5' RACE
Anchor primer2	GACCACGCGTATCGATGTCGAC	5' RACE
M13-F	GTAAAACGACGGCCAGT	5' RACE
esxA-QPCR-F	TGGCAATGATTAAGATGAGTCC	RT-qPCR
esxA-QPCR-R	TCTTGTTCTTGAACGGCATC	RT-qPCR
esaA-QPCR-F	TGGCTATAGAGCGAAATTCATC	RT-qPCR
esaA-QPCR-R	CCAAGCCTATAGGATGCTCTG	RT-qPCR
essC-QPCR-F	TTTCGATGTTGCAAGACACC	RT-qPCR
essC-QPCR-R	GACATGCGGAATTGTTTCAC	RT-qPCR
esxB-QPCR-F	GGTATTAAAGCAGATGGTGGCAAG	RT-qPCR
esxB-QPCR-R	GTCAGCCATCGGTTGTACTAATTC	RT-qPCR
16S rRNA-F	GTGCACATCTTGACGGTACCTA	RT-qPCR

16S rRNA-R	CCACTGGTGTTCCTCCATATC	RT-qPCR

Table S1. Oligonucleotide primers used in this study. Restriction enzyme sites are underlined.
